# Metabolism of Dopamine in Nucleus Accumbens Astrocytes Is Preserved in Aged Mice Exposed to MPTP

**DOI:** 10.3389/fnagi.2017.00410

**Published:** 2017-12-12

**Authors:** Brittany M. Winner, Harue Zhang, McKenzie M. Farthing, Lalitha M. Karchalla, Keith J. Lookingland, John L. Goudreau

**Affiliations:** ^1^Department of Pharmacology and Toxicology, Michigan State University, East Lansing, MI, United States; ^2^Institute for Integrative Toxicology, Michigan State University, East Lansing, MI, United States; ^3^College of Osteopathic Medicine, Michigan State University, East Lansing, MI, United States; ^4^Department of Neurology and Ophthalmology, Michigan State University, East Lansing, MI, United States

**Keywords:** nigrostriatal, mesolimbic, aging Parkinson disease, dopamine neurons, astrocytes

## Abstract

Parkinson disease (PD) is prevalent in elderly individuals and is characterized by selective degeneration of **n**igro**s**triatal **d**op**a**mine (NSDA) neurons. Interestingly, not all dopamine (DA) neurons are affected equally by PD and aging, particularly **m**eso**l**imbic (ML) DA neurons. Here, effects of aging were examined on presynaptic DA synthesis, reuptake, metabolism and neurotoxicant susceptibility of NSDA and mesolimbic dopamine (MLDA) neurons and astrocyte DA metabolism. There were no differences in phenotypic markers of DA synthesis, reuptake or metabolism in NSDA or MLDA neurons in aged mice, but MLDA neurons displayed lower DA stores. Astrocyte metabolism of DA to 3-methoxytyramine (3-MT) in the striatum was decreased in aged mice, but was maintained in the nucleus accumbens. Despite diminished DA vesicular storage capacity in MLDA neurons, susceptibility to acute neurotoxicant exposure was similar in young and aged mice. These results reveal an age- and neurotoxicant-induced impairment of DA metabolic activity in astrocytes surrounding susceptible NSDA neurons as opposed to maintenance of DA metabolism in astrocytes surrounding resistant MLDA neurons, and suggest a possible therapeutic target for PD.

## Introduction

Aging represents the greatest risk factor for certain neurodegenerative diseases including Parkinson disease (PD; Calne and William Langston, 1983). In PD, motor symptoms such as bradykinesia, resting tremor, postural instability, and shuffling gait (Gelb et al., 1999) occur due to loss of nigrostriatal dopamine (NSDA) neurons (Hornykiewicz, 1966) which are vital for the proper function of the basal ganglia pathway. Although NSDA neurons are lost over time as PD progresses, other dopaminergic neurons are relatively spared: tuberoinfundibular (TI) DA neurons and mesolimbic (ML) DA neurons resist degeneration (Braak and Braak, 2000). DA loss is more pronounced in the putamen of PD patients vs. other regions in the striatum (ST; Kish et al., 1988).

Mesolimbic dopamine (MLDA) neurons are often compared to NSDA neurons since they are phenotypically and anatomically similar, with soma in the ventral midbrain and axon terminals in the ventral striatum (Demarest and Moore, 1979). MLDA neurons in the ventral tegmental area (VTA) release DA in the nucleus accumbens (NAc) to function in circuits involved in motivation, reward and addiction. There is evidence that a link between the two DA systems in limbic control of motor activation exists (Mogenson et al., 1980), highlighting a potential interaction between NSDA and MLDA function. In a study aimed at comparing catecholamine uptake, [^3^H] mazindol uptake is decreased in both the ST and NAc of PD patients, with slightly more loss of [^3^H] mazindol uptake in the ST (−75%) compared to the NAc (−65%; Chinaglia et al., 1992). DA transport insufficiency in the NAc may partially explain why elderly patients with PD display deficits in reward processing (Schott et al., 2007), but does not explain why MLDA neurons do not degenerate.

Physiologically, both NSDA and MLDA neurons use DA as the key neurotransmitter, the latter of which has been linked to production of toxic DA adducts (Hastings et al., 1996; Caudle et al., 2007) and reactive oxygen species. Previous studies have examined differences between NSDA and MLDA neurons and how their dissimilarities translate into differential susceptibility between the two DA neuron populations (Surmeier et al., 2017). Despite the available understanding about factors that underlie the differential susceptibility of NSDA and MLDA neurons, few studies have focused on the role of aging. The studies with the neurotoxicant 1-methyl-4-phenyl-1,2,3,6-tetrahydropyridine (MPTP) have shown that aged mice demonstrate greater motor deficits and have more loss of DA in the ST when compared to younger mice following chronic MPTP exposure (Gupta et al., 1986). While neurotoxicant treated mice have limitations in modeling every aspect of PD, the MPTP model of DA depletion and oxidative stress can recapitulate differential susceptibility between NSDA and MLDA neurons (Hung and Lee, 1998; Behrouz et al., 2007). The differential susceptibility of MLDA and NSDA neurons observed in toxicant models has also been demonstrated in transgenic rodent models of PD-like pathology. In addition, studies in nonhuman primates have also recapitulated the resistance of VTA DA neurons to MPTP (Dopeso-Reyes et al., 2014). MLDA neurons are not susceptible to damage in rats after rAAV-mediated overexpression of α-synuclein in the ventral midbrain (Maingay et al., 2006), which indicates MLDA neurons are resistant to neurodegeneration elicited by distinct, but likely convergent etiologies of PD.

While these studies highlight intrinsic properties of MLDA neurons that enable survival in PD, there are non-neuronal cells in the cell body and nerve terminal region that likely play a role in differential responses to injury. While the production and metabolism of DA in the presynaptic DA neuron is well understood, the role of glial cells has only relatively recently been appreciated and contribution of astrocytes to diseases such as PD should be explored (Forno et al., 1992). Astrocytes express the catabolic enzymes monoamine oxidase B (MAO-B) and catechol-O-methyltransferase (COMT) and are the main non-neuronal cell type involved in the metabolism of DA (Levitt et al., 1982). Some studies suggest that astrocyte-mediated DA metabolism removes excessive synaptic DA, thus decreasing DA available for re-uptake, which would reduce cytoplasmic DA in the nerve terminal available for toxic DA adduct formation. Mice treated with chronic MPTP display abnormal proliferation of astrocytes in the ST, a process known as astrogliosis (Dervan et al., 2004).

If astrocytes safeguard MLDA neurons in aging and under neurotoxic stress, therapeutic strategies to promote protective functions of astrocytes in regions affected by PD could be developed. The involvement of astrocytes in age-related decline of DA neuronal function in the NAc has not yet been explored. To this end, we have conducted a series of experiments in various ages of mice to determine if DA synthesis, release and metabolism are altered in MLDA neuronal axon terminals or astrocytes from aged mice and whether MLDA neurons are rendered susceptible to MPTP exposure with aging. We hypothesize that MLDA neurons are resistant to the effects of toxicant exposure in both young and aged mice and that uptake and metabolism of DA by astrocytes is preserved in the NAc of aged mice. Our study addresses the contribution of axon terminals and surrounding astrocytes to metabolism of DA in the aged NAc vs. the ST to better understand differences in MLDA and NSDA neuronal and astrocyte susceptibility to aging.

## Materials and Methods

### Chemicals and Antibodies

Unless otherwise specified, all chemicals and reagents were purchased from Sigma-Aldrich. Rabbit anti-tyrosine hydroxylase (TH) primary antibody was purchased from Millipore (MAB152, AB_390204) and used at a 1:2000 dilution. Rabbit anti-Ser40 p-TH was purchased from Cell Signaling (#2791, AB_2201522) and used at a 1:1000 dilution. Rat anti-DAT was purchased from Millipore (MAB369, AB_2190413) and used at a 1:1000 dilution. Rabbit anti-VMAT2 was purchased from Millipore (AB1598P, AB_2285927) and used at a dilution of 1:1000. Rabbit anti-COMT was purchased from Abcam (ab126618, AB_11129919) and used at a dilution of 1:4000. Mouse anti-GAPDH was purchased from Sigma Aldrich (G8795, AB_1078991) and used at a dilution of 1:4000. All primary antibodies were diluted in 5% BSA in TBS-T. Secondary antibodies linked to horse radish peroxidase were purchased from Cell Signaling (rabbit: #7074s; mouse: #7076; rat: #7077) and diluted to 1:4000 before use in 5% nonfat milk in TBS-T.

### Animals and Drug Treatment

GFP-TH mice expressing GFP under the control of the rat TH promoter (Sawamoto et al., 2001) were bred in-house and divided into four age groups: young (3–8 months, *n* = 19); mature (9–12 months, *n* = 28); old (12.5–14 months, *n* = 23); and aged (22–28 months, *n* = 20). Mice were housed in a temperature (22 ± 1°C) and light controlled (12 h light/dark cycle) room and provided with food and water *ad libitum*. This study was carried out in accordance with the recommendations of the Michigan State University Institutional Animal Care and Use Committee, which approved all experiments using live animals (AUF 10/14-183-00). MPTP hydrochloride was obtained from Santa Cruz Inc., dissolved in saline, and the dose was calculated as the free base (Jackson-Lewis and Przedborski, 2007). Mice were randomly assigned to treatment groups and received a single s.c. injection of either 10 mL/kg saline vehicle or 20 mg/kg MPTP. Twenty-four hours after injection, mice were decapitated and brains sectioned using a brain matrix (Zivic Instruments) to a 1.0 mm thick slice. ST and NAc were microdissected using an 18 gauge needle following anatomical tissue structure identification via a stereotaxic brain atlas (Paxinos and Franklin, 2004) and tissue samples were frozen and stored at −80°C until use. Endpoint assays were performed blinded to treatment group.

### Neurochemical Analyses via HPLC-ED

Microdissected samples of ST and NAc were prepared for neurochemical analysis as previously described (Benskey et al., 2012). Sample content of DA, 3,4-hydroxyphenylacetic acid (DOPAC), homovanillic acid (HVA) and 3-methoxytyramine (3-MT) was determined by high pressure liquid chromatography coupled with electrochemical detection (HPLC-ED) and the concentration of neurochemicals was expressed in nanogram (ng) per milligram (mg) protein. Protein content per sample was determined via bicinchoninic acid assay.

### Immunoblot Analyses

Micropunch samples of ST and NAc were processed and Western blots were performed as previously described (Benskey et al., 2012). Protein bands were visualized using a PICO or FEMTO Chemiluminescence substrate (ThermoFisher) kit on a LI-COR Odyssey imager. Bands from each lane of the protein of interest were normalized to the loading control GAPDH using Image Studio version 5.2.

### Statistical Analysis

All statistical analyses were performed using SigmaPlot v. 12 software. For experiments containing two factors (age and treatment), a two-way ANOVA was performed with a *post hoc* Holm-Sidak test to determine if there is a statistically significant difference between groups (*p* < 0.05). In studies where only one factor is examined (age), a one-way ANOVA was performed with a *post hoc* Holm-Sidak test to determine if there is a statistically significant difference between groups (*p* < 0.05). At least six mice per sample group were used for each analysis to yield a power of ≥80%. Outliers in each experimental group were removed before statistical analysis using a Grubbs outlier test^1^ for significance level *p* < 0.05. All graphical representations of data denote the mean + SEM.

## Results

### Aged Mice Have Less Stored DA in the NAc, but Astroglial Metabolism of DA and COMT Expression Remain Intact

**Figure 1:**
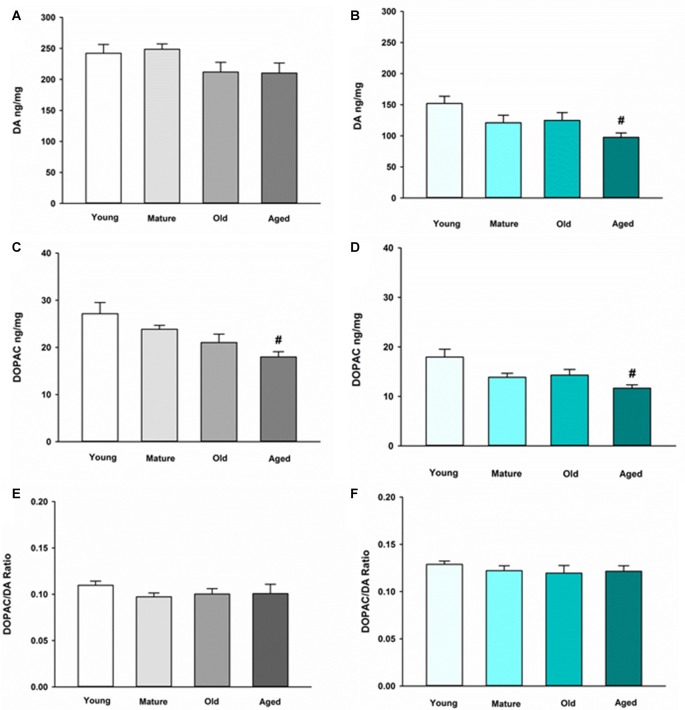
Dopamine (DA) and 3,4-dihydroxyphenylacetic acid (DOPAC) concentrations and DOPAC/DA ratio in striatum (ST) **(A,C,E)** and nucleus accumbens (NAc) **(B,D,F)** in Young, Mature, Old and Aged mice. Young, Mature, Old and Aged mice were decapitated, brains were sectioned, and ST and NAc were collected in tissue buffer for high pressure liquid chromatography coupled with electrochemical detection (HPLC-ED) analysis. DA and DOPAC were measured and normalized to mg protein per sample. Concentrations for DOPAC were divided by DA to give the DOPAC/DA ratio. Data is expressed as mean + SEM. Data was analyzed via one-way ANOVA with *post hoc* Holm-Sidak test. Hash symbol (#) indicates statistically significant difference (*p* < 0.05) from Young age group.

We first sought to determine the basal neurochemical profile for DA in the NAc and ST in young, mature, old and aged mice. In the ST, DA was not altered in any of the various age groups compared to young control (Figure 1A). In the NAc, however, DA is decreased in aged mice compared to young mice (Figure 1B). The main metabolite of recaptured DA by MAO-B, DOPAC, also decreased in an age-dependent manner in the ST (Figure 1C) and the NAc (Figure 1D). The DOPAC/DA ratio is a measure of the relative rate of DA metabolism via MAO-B compared to release, uptake and storage in synaptic vesicles. The DOPAC/DA ratio is not changed in the ST (Figure 1E) or the NAc between age groups (Figure 1F). This latter observation indicates that the rate of DA metabolism relative to storage and release of recaptured DA from the synapse is not affected by aging in DA axon terminals in either brain region.

**Figure 2:**
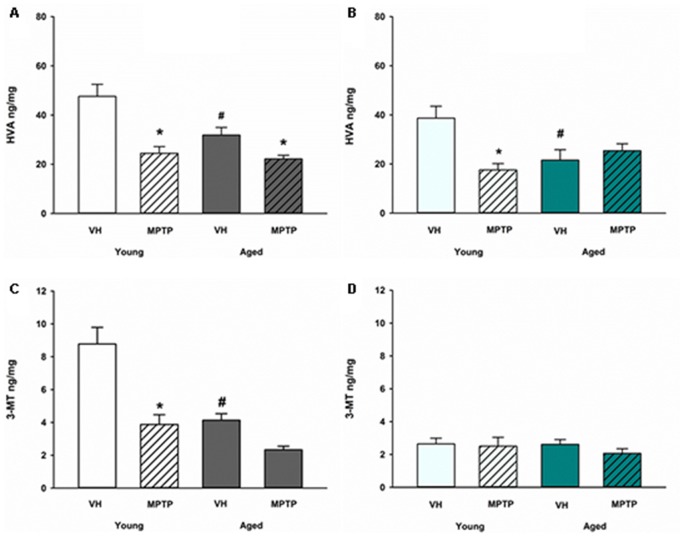
Homovanillic acid (HVA) and 3-methoxytyramine (3-MT) concentrations after 1-methyl-4-phenyl-1,2,3,6-tetrahydropyridine (MPTP) in Young and Aged ST **(A,C)** and NAc **(B,D)**. Young and aged mice were treated with vehicle (VH) or 20 mg/kg MPTP and sacrificed 24 h later. Brains were sectioned and ST and NAc were collected in tissue buffer for HPLC-ED analysis. HVA and 3-MT were measured and normalized to mg protein per sample. Data is expressed as mean + SEM. Data was analyzed via two way ANOVA with *post hoc* Holm-Sidak test. Asterisk (*) indicates statistically significant difference (*p* < 0.05) from VH control, while hash symbol (#) represents statistically significant difference from Young VH.

**Figure 3:**
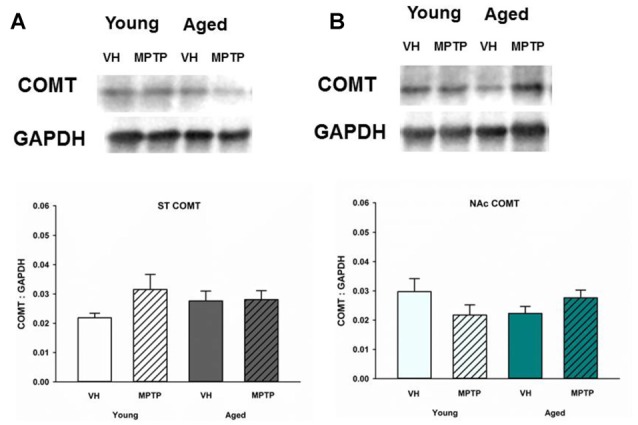
Catechol-O-methyltransferase (COMT) protein levels in the ST **(A)** and NAc **(B)** Young and aged mice were treated with VH or 20 mg/kg MPTP and sacrificed 24 h later. Brains were sectioned and ST and NAc were collected in lysis buffer for Western blot analysis. After gel electrophoresis, membranes were probed for COMT and densitometry was performed to normalize each band to the GAPDH loading control. Representatives of immunoblots are shown in figure. Data is expressed as mean + SEM and analyzed via two way ANOVA with *post hoc* Holm-Sidak test. No statistically significant difference (*p* < 0.05) was detected from VH control.

Extraneuronal metabolism of DA released from the synaptic nerve terminal produces HVA and 3-MT. To determine if age alters extraneuronal metabolism of released DA, HVA and 3MT were measured in young and aged mice (Figure 2). HVA was decreased with age in both the ST (Figure 2A) and the NAc (Figure 2B). 3-MT, however, was decreased with age in the ST (Figure 2C) but not in the NAc (Figure 2D). These results suggest that extraneuronal astrocyte metabolism of DOPAC to HVA by COMT or DA to 3-MT by MAO-B could be compromised in aged mice in the ST. In contrast, the extraneuronal (astroglial) metabolism of released DA does not appear to be diminished in the NAc as mice get older. We measured total COMT protein in the ST (Figure 3A) and NAc (Figure 3B) in young and aged mice and found no significant difference between age groups.

### Acute MPTP Exposure Decreases DA, DOPAC and Astroglial DA Metabolites in ST and NAc of Both Young and Aged Mice

**Figure 4:**
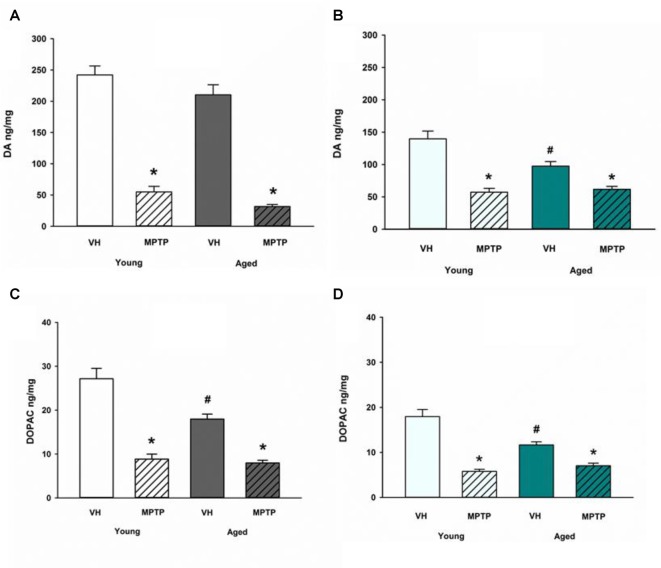
DA and DOPAC concentrations after MPTP in Young and Aged ST **(A,C)** and NAc **(B,D)**. Young and aged mice were treated with VH or 20 mg/kg MPTP and sacrificed 24 h later. Brains were sectioned and ST and NAc were collected in tissue buffer for HPLC-ED analysis. DA and DOPAC were measured and normalized to mg protein per sample. Data is expressed as mean + SEM. Data was analyzed via two way ANOVA with *post hoc* Holm-Sidak test. Asterisk (*) indicates statistically significant difference (*p* < 0.05) from respective VH control within age group, while hash symbol (#) represents statistically significant difference from Young VH control.

Next, we challenged mice with the neurotoxicant MPTP to determine if loss of DA axonal stores in the NAc observed after MPTP exposure is exacerbated in aged mice. Aged mice are not more sensitive to acute MPTP-induced loss of DA in the ST (Figure 4A) but aged mice are less susceptible to loss of DA in the NAc compared to young mice (Figure 4B). Loss of DOPAC after MPTP is equivalent between age groups in the ST (Figure 4C) but the MPTP-induced decrease in DOPAC is reduced in the NAc of aged mice (Figure 4D). The diminished MPTP-induced loss of DA and DOPAC in the NAc aged mice compared to young mice may reflect the combined effects of age-dependent and MPTP-induced loss of DA and DOPAC. There may also be a lower limit to the extent of DA and DOPAC loss that can be achieved with a single, acute 20 mg/kg dose of MPTP. This lower limit for toxicant-induced DA impairment may represent a resilient population of NAc DA neurons in advanced age.

A differential effect of MPTP was also observed in the astroglial metabolism of DA in the MLDA and NSDA regions. HVA, a direct metabolite of DOPAC, decreases in the ST of both young and old mice following acute MPTP treatment (Figure 2A). In the NAc, however, HVA decreases in young mice treated with MPTP (Figure 2B), but is not further decreased in aged mice treated with MPTP. Following MPTP treatment, the COMT DA metabolite 3-MT decreased in the ST of both young and aged mice (Figure 2C) but was unchanged in the NAc of young or aged mice (Figure 2D). The unique MPTP-induced changes in HVA and 3MT in the ST and NAc of young and aged mice suggests that extraneuronal metabolism of DOPAC and DA in astrocytes are differentially regulated in these two brain regions in the context of acute neurotoxic stress.

### Total and Phosphorylated TH Are Decreased in the ST but Not the NAc after MPTP in Young and Aged Mice

**Figure 5:**
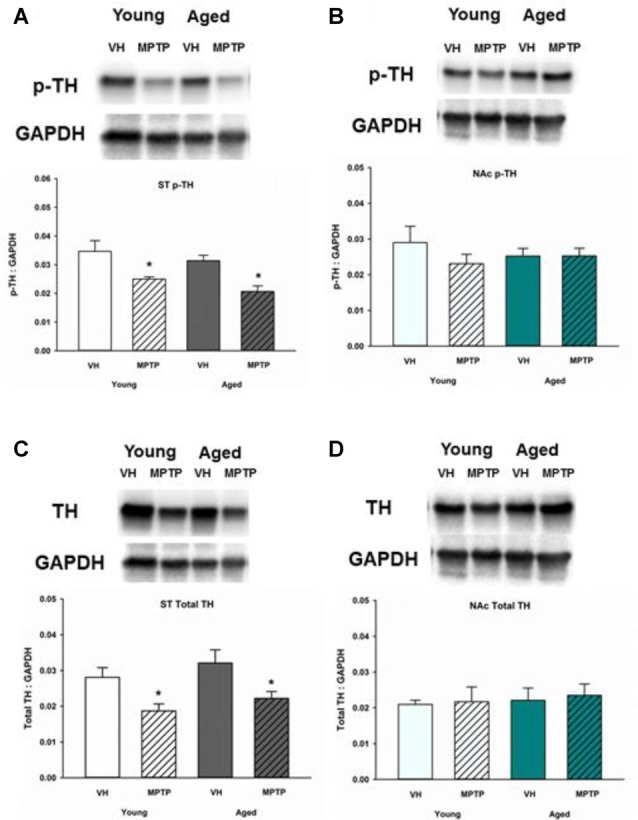
Ser40 phosphorylated tyrosine hydroxylase (p-TH) and total TH protein levels in ST **(A,C)** and NAc **(B,D)**. Young and aged mice were treated with VH or 20 mg/kg MPTP and sacrificed 24 h later. Brains were sectioned and ST and NAc were collected in lysis buffer for Western blot analysis. After gel electrophoresis, membranes were probed for Ser40 p-TH and TH and densitometry was performed to normalize each band to the GAPDH loading control. Representatives of immunoblots are shown in figure. Data is expressed as mean + SEM and analyzed via two way ANOVA with *post hoc* Holm-Sidak test. Asterisk (*) indicates statistically significant difference (*p* < 0.05) from VH control.

Given the observed age-related decrease of stored DA in the NAc, we sought to determine if synthesis and vesicular storage of DA are also decreased in the NAc and ST of aged mice compared to younger mice. Total TH and Ser40 phosphorylated TH (p-TH) were measured in the NAc (Figure 5). TH is the rate-limiting enzyme of DA synthesis and requires phosphorylation of serine residue 40 to become active (Campbell et al., 1986). Both Ser40 p-TH and total TH were decreased in the ST following MPTP treatment in young and aged mice (Figures 5A,C). In the NAc, however, total TH and Ser40 p-TH expression was similar in saline and MPTP treated young and aged mice (Figures 5B,D). Therefore, the MPTP-induced decrease in DA observed in the NAc is not due to a deficit in total or activated TH available for DA synthesis.

### DAT Is Decreased in the ST but Not the NAc after MPTP While VMAT2 Is Unchanged in Both Young and Aged Mice Treated with MPTP

**Figure 6:**
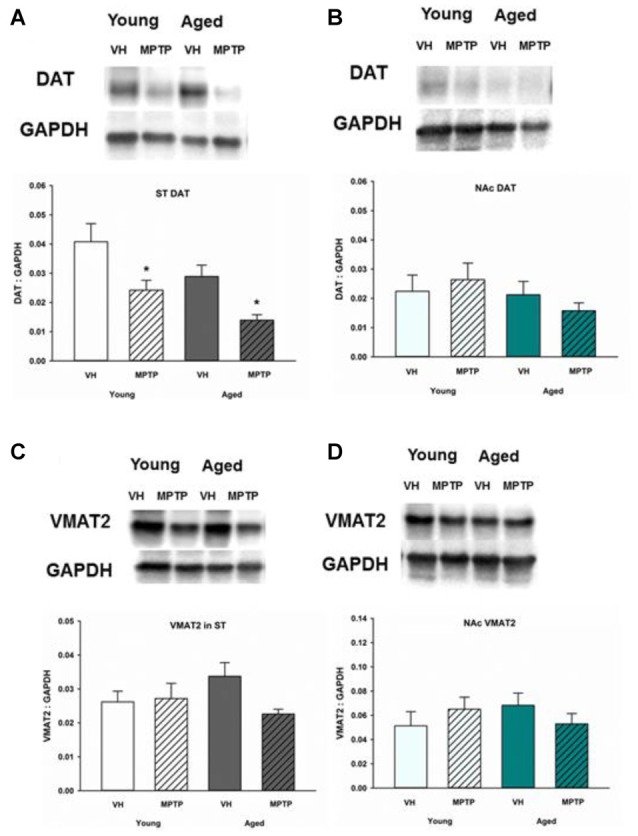
Dopamine transporter (DAT) and vesicular monoamine transporter 2 (VMAT2) protein levels in ST **(A,C)** and NAc **(B,D)**. Young and aged mice were treated with VH or 20 mg/kg MPTP and sacrificed 24 h later. Brains were sectioned and ST and NAc were collected in lysis buffer for Western blot analysis. After gel electrophoresis, membranes were probed for DAT and VMAT2 and densitometry was performed to normalize each band to the GAPDH loading control. Representatives of immunoblots are shown in figure. Data is expressed as mean + SEM and analyzed via two way ANOVA with *post hoc* Holm-Sidak test. Asterisk (*) indicates statistically significant difference (*p* < 0.05) from VH control.

To determine if reuptake or vesicular storage of DA could be affected by aging in the ST and NAc, DAT and vesicular monoamine transporter 2 (VMAT2) were measured. We observed no change in levels of these proteins between young and aged mice in either brain region (Figures 5, 6). Cytoplasmic DAT is susceptible to oxidative damage and toxic adduct formation. In addition to synthesis and metabolism, cytoplasmic DA concentrations are regulated by the relative capacity for DA uptake via DAT and vesicular storage via VMAT. In addition to recycling synaptic DA, DAT is also the transporter through which the active MPTP metabolite, MPP^+^ enters into DA neurons and astrocytes. Relative DAT levels reflect the axon terminal DA uptake capacity. As such, measuring DAT expression can assess potential mechanisms for age and region specific differential susceptibility to MPTP and oxidative stress. DAT decreases in the ST following acute MPTP treatment (Figure 6A). In contrast, DAT expression in the NAc is similar in vehicle and MPTP treated mice (Figure 6B). VMAT2 packages DA into synaptic vesicles and reduces the amount of cytoplasmic DA available for oxidative conversion to toxic molecules (e.g., DA quinones). VMAT2 was not altered in either brain region in saline and MPTP treated young or aged mice (Figures 6C,D).

## Discussion

### DA Synthesis, Metabolism, Reuptake and Packaging in Aged Presynaptic Axon Terminals

**Figure 7:**
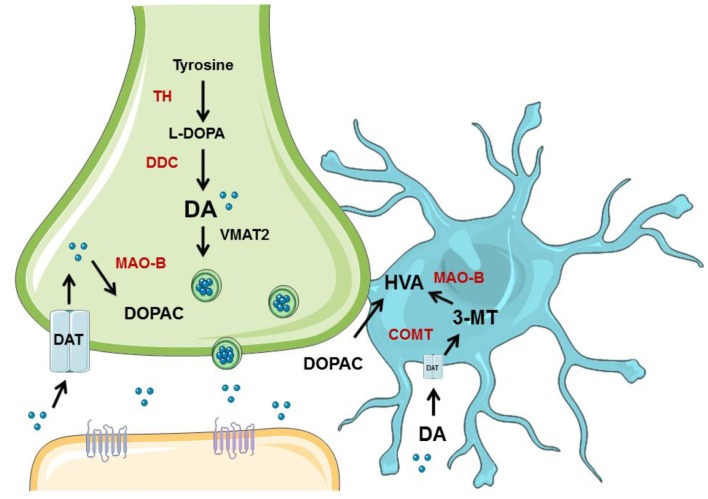
Overview of neuronal synthesis of DA and astrocyte metabolism. DA synthesis occurs in a presynaptic neuron (green) and is metabolized or packaged into vesicles and released into the synapse. DA is synthesized from tyrosine by TH to L-DOPA, which is then metabolized to DA via dopa decarboxylase (DDC). DA can then be metabolized by monoamine oxidase (MAO) to DOPAC, which can be exported to astrocytes (blue) for further metabolism into HVA by COMT. If DA is packaged into vesicles by VMAT2, it can be released into the synapse and bind to receptors on the postsynaptic neuron (orange). DA can also be transported back into the axon terminal by DAT. If DA is exported to the astrocyte, it can undergo metabolism by COMT to 3-MT, which then can be metabolized by MAO to HVA. Graphics were adapted from Servier Medical Art (www.servier.com) with a Creative Commons Attribution 3.0 Unported License and edited for use in this figure.

Young animals are frequently used in experiments designed to understand PD, yet have clear limitations in the study of a disease so closely associated with aging. Indeed, recent evidence suggests that DA neuron degeneration observed in normal aging occurs through similar, convergent mechanisms as DA neuron degeneration in PD (Collier et al., 2011). Many studies have been published that show that NSDA neurons degenerate with age (Stark and Pakkenberg, 2004), but few studies compare aged NSDA and MLDA neurons, much less in terms of function. We sought to evaluate age-related changes in two groups of midbrain DA neurons differentially affected in PD under basal conditions and following toxicant exposure. Presynaptic and extraneuronal DA metabolic pathways were assessed in the brain regions containing the nerve terminals of the NSDA and MLDA neurons, the ST and NAc, respectively (Figure 7).

First, we determined if presynaptic DA metabolism and release are perturbed in aged mice. After investigating changes in DA concentrations between the ST and NAc, we observed that DA was not changed with age in the ST but was decreased in aged mice in the NAc. We examined levels of total and Ser40 p-TH to determine if the shortage of DA stores could be linked to deficient availability of the active form of the rate-limiting enzyme essential for DA synthesis. We did not, however, observe an age-related decrease in p-TH or total TH in the NAc. As such, it appears unlikely that impaired DA synthesis capacity is an explanation for the age-related decrease in MLDA stores.

If synthesis of DA was unperturbed in aged NAc, then perhaps reuptake of DA and presynaptic metabolism of DA to DOPAC increases with age, thus explaining the age-dependent decrease in MLDA stores. Corroborating our finding that aging does not affect total DAT levels in either brain region, a previous study reported no difference in total DAT levels with aging in rat ST (Cruz-Muros et al., 2009). Aged mice were found to have less DOPAC in the NAc than young mice in our study. The majority of DOPAC is produced when DA is taken back up into the axon terminal (Roth et al., 1976) and metabolized by MAO-B. Thus DOPAC represents an indirect index of DA that has been released and recaptured by the presynaptic neuron. Our observation that aged mice have lower DOPAC concentrations compared to young mice suggests that MLDA neurons in aged mice release less DA or metabolize less intraneuronal cytoplasmic DA. One study found that aged rats release less DA in the NAc as measured by microdialysis, which is congruent with our results (Huang et al., 1995). DA release is dependent on DA neuronal activity. The ratio of DOPAC/DA is an index of DA metabolism or turnover that is independent of DA nerve terminal density (Lookingland and Moore, 2005). Notably, the ratio of DOPAC/DA was similar in the NAc of young and aged mice, which suggests that there is no age-related increase in DA turnover in MLDA neurons that could lead to accumulation of toxic metabolites and loss of axon terminals as seen in NSDA neurons terminating in the ST.

Changes in DA uptake or synaptic vesicular storage capacity, in part, regulate the pool of cytoplasmic DA that is susceptible to oxidative conversion to toxic molecules (Hastings et al., 1996). Unregulated cytosolic DA was sufficient to cause neurodegeneration in one study using transgenic mice (Chen et al., 2008), highlighting the important role of modulating cytosolic DA. To understand whether reuptake or vesicular packaging could affect the concentrations of DA or DOPAC, we measured DAT and VMAT2 in ST and NAc after aging. No significant alterations in DAT and VMAT2 were found in aged mice in either brain region. As such, presynaptic reuptake and synaptic vesicle compartmentalization of DA are not affected by aging in the NAc.

### DOPAC and DA Metabolism in Aged Astrocytes

Astrocytes are expected to play a major protective role in regulating synaptic DOPAC and DA metabolism in regions containing DA neurons. However, although other studies measured numbers of astrocytes and microglia in aged mouse brain tissue such as the hippocampus, finding that aging does not alter the total number of glial cells (Long et al., 1998), the main effort in our study was to measure functional capability of astrocytes in DAergic brain regions in aged mice. We therefore assessed extraneuronal metabolism of DA by astrocytes. If changes in presynaptic DA synthesis, release-reuptake and intraneuronal metabolism cannot explain the age-dependent decline in MLDA stores, then alterations in extraneuronal DA metabolism may play a role. Impairment of astrocytes have been reported to contribute to alterations in DA metabolism and progression of PD (Maragakis and Rothstein, 2006). COMT, present only within astrocytes metabolizes DOPAC to HVA. Measurement of HVA, therefore, provides an index of glial metabolism of extraneuronal DOPAC (Schendzielorz et al., 2013). Both the ST and NAc experienced an age-related decrease in HVA, which suggests that less DOPAC is being metabolized into HVA in astrocytes of aged mice.

The decrease in HVA could reflect less substrate (DOPAC) available, decreased glial COMT expression, or a decrease in the number of glial cells expressing COMT. COMT protein levels were not decreased in the ST or NAc of aged mice. Also, a prior study by Emsley and Macklis (2006) reported there is approximately the same density of astrocytes in the ST and NAc (Emsley and Macklis, 2006), which is supported by our COMT protein expression data. Another DA metabolite, 3-MT, is the direct product of metabolism of DA by COMT in astrocytes and represents an indirect index of DA release (Figure 6). Since 3-MT was not decreased with aging in the NAc, there could be an increase in activity of glial COMT to compensate for decreased substrate. Our results indicate that astrocytes retain expression of COMT protein and the ability to metabolize DA in the NAc in aged mice.

### Acute MPTP Exposure in Aged Mice

Toxicant induced oxidative damage results in a decrease in DA in the ST and NAc, in addition to loss of axon terminals of NSDA and MLDA neurons. Not only MPTP has been used in this context: an earlier study from our laboratory using chronic rotenone in rats (Behrouz et al., 2007) recapitulated the selective vulnerability of the NSDA neurons compared to less vulnerable MLDA neurons. In addition, 6-OHDA causes a more severe lesion in the SN compared to the VTA, as quantified by stereology (Grealish et al., 2010). Susceptibility to axon terminal loss and cell body degeneration in NSDA neurons may increase with age (McGeer et al., 1977; Gupta et al., 1986; Stark and Pakkenberg, 2004). We specifically used the acute MPTP dosing paradigm to allow investigation of DA axon terminal and local astrocyte responses in the absence of significant neuronal loss or denervation. Early events occurring following MPTP-induced injury predispose neurons to eventual degeneration but also may represent potentially reversible steps in the process that may be more amenable to disease modifying therapeutic intervention.

We observed that decrease in DA caused by a single acute dose of MPTP was similar in NSDA and MLDA neurons in young and aged mice. With chronic, repeated exposure to MPTP, the susceptibility of NSDA neurons does appear to increase with age (Gupta et al., 1986; Ricaurte et al., 1987; Date et al., 1990). Chronic, repeated MPTP treatment eventually results in both ST axon terminal and SN cell body loss, while a single acute MPTP treatment causes axon terminal dysfunction but does not produce cell death at the time periods examined in the present study.

DAT mediates the uptake of MPP^+^ and DAT expression is required for MPTP toxicity (Gainetdinov et al., 2002). DAT expression depends, to a large extent, on the number of DA axon terminals, but the overall and surface expression of DAT can change independent of the density of DA nerve terminals (Nirenberg et al., 1996). Total DAT expression decreases to a similar extent in the ST after acute MPTP treatment in both young and aged mice. DAT expression was similar in saline and MPTP treated mice in the NAc, regardless of age. As previously noted, the acute MPTP paradigm we employ does not result in significant NSDA axon terminal loss (Jackson-Lewis et al., 1995). Indeed, we observed no significant change in the expression a more stable marker of DA nerve terminal density (VMAT2; Vander Borght et al., 1995) following acute MPTP treatment. Mice heterozygous mutants for DAT or VMAT2 experienced age-related degeneration in the SN (Hall et al., 2014), suggesting that maintenance of DAT and VMAT2 levels may help protect SN neurons. Our findings, therefore, suggest that the early and likely dynamic decline in ST DAT expression following acute neurotoxicant injury is not age dependent. Our results also provide some reassurance that capacity for MPP^+^ uptake via DAT does not change significantly with age and is not a likely confounding factor for our observations. However, it is still possible that aged mice metabolize MPTP to MPP^+^ at a different rate compared to young mice. One study found that aged mice have higher amounts of MAO-B in brain (Saura et al., 1994), suggesting that at least expression levels are different between age groups. However, another study found that young and older mice have similar concentrations of MPP+ after MPTP injection, indicating that increased bioactivation of MPTP to MPP^+^ is not the causal mechanism for age-related increase in susceptibility (Ricaurte et al., 1987).

It should be noted that measurement of total DAT protein expression does not always parallel the DAT localized or expressed on the plasma membrane surface. This is an important caveat, since the surface expression of DAT determines the true uptake capacity of this transporter (Mortensen and Amara, 2003). Cytoplasmic DA available for oxidative conversion to more toxic molecules would be expected to be low in brain regions with high storage capacity (VMAT2) relative to re-uptake (DAT; Uhl, 1998). Basal expression of VMAT2 or the ratio of VMAT2/DAT are inversely correlated with the susceptibility of different brain regions to MPTP (Hall et al., 2014; Lohr et al., 2016). VMAT2 expression was not changed in either brain region in young or aged mice and also did not change following acute MPTP treatment. This observation indicates there was no age-dependent change in the capacity of NSDA and MLDA axon terminals to store DA in synaptic vesicles. Direct or indirect measurement of VMAT2 is closely linked to nerve terminal density. Since the acute MPTP model we employed in the current study does not cause significant cell body or axon loss, then it is not surprising there was no observed change in VMAT2 following MPTP treatment.

Taken together, the above findings indicate alterations in pre-synaptic DA metabolic homeostasis do not change with age in the NAc and are not likely to explain the age-dependent decline in NAc DA stores. Age-dependent changes in astrocyte metabolism of extraneuronal DA, however, may play a role. This is evidenced by impairment of extraneuronal metabolism of DA to 3-MT, but not the NAc, and that HVA produced by the metabolism of DOPAC via COMT and the conversion of 3-MT by MAO, decreases in both the ST and NAc with age.

Since DAT is expressed in astrocytes as well as presynaptic neurons (Schömig et al., 1997; Takeda et al., 2002), MPP^+^ can be taken up by astrocytes and cause oxidative damage. In the NAc, however, no decrease in 3-MT was observed in mice exposed to MPTP. These results suggest that astrocytes could be playing a protective role in the NAc. Indeed, under conditions of heightened oxidative stress, astrocytes may be overwhelmed and no longer able to properly protect ST DA neurons (Episcopo et al., 2013). Perhaps exceeding the protective capacity of glial cells contributes to neurodegeneration in PD. Consistent with this hypothesis, astrocytes are activated in regions containing NSDA neurons in younger primates treated with MPTP, but this activation is attenuated in aged primates and is associated with an increased impairment of striatal DA metabolism (Kanaan et al., 2008). In addition, the same study showed that astroglial markers were not altered in NSDA or MLDA brain regions from MPTP-treated primates compared to aged controls (Kanaan et al., 2008). In the present study astrocyte metabolism is relatively preserved in the NAc of aged mice and this maintained capacity for extraneuronal DA metabolism may stabilize DA homeostasis by rapidly clearing synaptic DA in MLDA neurons under conditions of oxidative stress-induced injury.

## Conclusion

This study was designed to test the hypothesis that MLDA neurons are less susceptible to age-related deficits in DA synthesis, metabolism and reuptake compared to well-characterized NSDA neurons. We measured the components of presynaptic axon terminal and astrocyte DA synaptic homeostasis including metabolites, synthetic enzymes, and transporters to understand the impact of aging on DA homeostasis in MLDA and NSDA neurons. We also exposed young and aged mice to acute neurotoxic insult to examine how aged MLDA neurons cope with neurotoxicant-induced alterations in DA metabolism. We conclude that DA and DOPAC loss in the NAc is consistent with a decrease in release of DA while DA synthesis remains unaffected. As contributors to metabolism of DA released in the synapse, astrocytes in the NAc retain the ability to metabolize DA into 3-MT, suggesting these cells are capable of fulfilling their neuroprotective role in both young and aged mice. Our study highlights the importance of DA metabolism in astrocytes and the potential role of astrocytes in contributing to the differential susceptibility of NSDA and MLDA neurons in PD.

## Author Contributions

BMW designed, performed and analyzed experiments; HZ and MMF analyzed the data and approved the final manuscript; LMK helped perform the experiments and approved the final manuscript; KJL and JLG helped design the experiments and approved the final manuscript.

## Conflict of Interest Statement

The authors declare that the research was conducted in the absence of any commercial or financial relationships that could be construed as a potential conflict of interest.

## Footnotes

^1^www.graphpad.com/quickcalcs/Grubbs1.cfm

## Abbreviations

COMT: catechol-O-methyltransferase
DA: dopamine
DAT: dopamine transporter
DOPAC: 3,4-dihydroxyphenylacetic acid
HVA: homovanillic acid
MLDA: mesolimbic dopamine
NAc: nucleus accumbens
NSDA: nigrostriatal dopamine
PD: Parkinson disease
ST: striatum
SN: substantia nigra
TH: tyrosine hydroxylase
VMAT2: vesicular monoamine transporter
VTA: ventral tegmental area
3-MT: 3-methoxytyramine.
